# Rotavirus NSP4_114-135 _peptide has no direct, specific effect on chloride transport in rabbit brush-border membrane

**DOI:** 10.1186/1743-422X-3-94

**Published:** 2006-11-13

**Authors:** Mathie Lorrot, Monique Vasseur

**Affiliations:** 1 Hôpital Robert Debré, Service de Pédiatrie Générale, Paris, F-75019, France; 2 INSERM, UMR 756, Université de Paris XI, Faculté de Pharmacie, Châtenay-Malabry, F-92296, France

## Abstract

The direct effect of the rotavirus NSP4_114-135 _and Norovirus NV_464-483 _peptides on ^36^Cl uptake was studied by using villus cell brush border membrane (BBM) isolated from young rabbits. Both peptides inhibited the Cl^-^/H^+ ^symport activity about equally and partially. The interaction involved one peptide-binding site per carrier unit. Whereas *in vitro *NSP4_114-135 _caused nonspecific inhibition of the Cl^-^/H^+ ^symporter, the situation *in vivo *is different. Because rotavirus infection in young rabbits accelerated both Cl^- ^influx and Cl^- ^efflux rates across villi BBM without stimulating Cl^- ^transport in crypt BBM, we conclude that the NSP4_114-135 _peptide, which causes diarrhea in young rodents, did not have any direct, specific effect on either intestinal absorption or secretion of chloride. The lack of direct effect of NSP4 on chloride transport strengthens the hypothesis that NSP4 would trigger signal transduction pathways to enhance net chloride secretion at the onset of rotavirus diarrhea.

## Findings

Rotavirus is the major cause of infantile gastroenteritis and each year causes 611000 deaths worldwide. A rotavirus nonstructural glycoprotein, NSP4, and a synthetic peptide, NSP4_114-135_, corresponding to residues 114 to 135 of this protein, both have been shown to induce diarrhea in young rodents, unaccompanied by any histological lesions [1]. But despite considerable research over several decades, the mechanisms underlying the diarrheal illness remain unclear [2,3].

The rotavirus NSP4_114-135 _peptide has been shown to interact with small unilamellar phospholipid vesicles characterized by highly curved membrane regions [4]. However, it is unknown whether such interaction of NSP4 with a putative membrane receptor may be important for its biological activity. Tian et al. reported that NSP4 and NSP4_114-135 _caused membrane destabilization activity [5]. This seems to be true for liposomes and endoplasmic reticulum vesicles, but not for plasma membrane vesicles such as intestinal brush border membrane vesicles (BBM). On the other hand, the NSP4_114-135 _peptide has been shown to directly and specifically inhibit the SGLT1-mediated Na^+^-D-glucose symport activity in villi BBM of rabbit intestine [6]. In contrast, the Norovirus NV_464-483 _and mNSP4_131K _(NSP4_114-135 _having an L-lysine residue substituting for the L-tyrosine at position 131) peptides neither cause diarrhea nor inhibit SGLT1. The selective and strong inhibition caused in vitro by NSP4_114-135 _on SGLT1 suggests that, during rotavirus infection *in vivo*, the newly synthesized glycoprotein NSP4 is released into the intestinal lumen and acts on the SGLT1 protein, hence, directly causing glucose malabsorption and a concomitant inhibition of water reabsorption [7].

The observation that addition of either NSP4 or carbachol (a cholinergic agonist that mobilizes Ca^2+^) to neonatal mouse intestinal mucosal sheets induced transient, small and almost identical increases in Cl^- ^secretory currents was interpreted as indicating that NSP4 induced a Ca^2+^-dependent Cl^- ^secretory mechanism [1]. However, the cellular and molecular bases by which rotavirus and NSP4 induce a moderate net chloride secretion remain unclear. Recently, Lorrot et al. reported that rotavirus infection *in vivo *in young rabbits failed to stimulate the Cl^- ^transport activities at the crypt level, but not at the villus level, questioning, therefore, the origin of net chloride secretion at the onset of diarrhea [8,9]. Because rotavirus stimulated both Cl^- ^influx and Cl^- ^efflux in villi, Lorrot et al. proposed that the Cl^-^/H^+ ^symporter might function in both normal (absorption) and reversed (secretion) modes, depending on the direction of the chloride electrochemical gradient resulting from rotavirus infection [9].

In the present study, we examined whether or not the ability of rotavirus to stimulate chloride transport across rabbit villus cell BBM might be due to the direct activity of NSP4_114-135_. The Norovirus NV_464-483 _peptide was tested as a possible control since this peptide, unlike NSP4_114-135_, does not cause diarrhea even though its amphipathic score is practically identical to that of NSP4_114-135 _[1].

Both peptides were the gift of Dr. J. M. Ball (College of Veterinary Medicine and Biomedical Sciences, Texas A&M University, College Station, Texas) and Dr. M. K. Estes (Baylor College of Medicine, Houston, Texas). Because of peptide solubility and the inevitable carry-over of a quantity of peptide from the preincubation to the incubation media (in the proportion of 1/10), the maximum peptide concentration that could be reached in the incubation mixtures was 0.55 mM [6]. The NSP4 protein action could not be demonstrated in the present paper, mainly because the maximum concentration of 0.5 μM was found to be too low to significantly affect chloride uptake [6]. Intestinal villi BBM vesicles were prepared from specific pathogen-free, four-week-old New Zealand albino hybrid rabbit by using the magnesium precipitation method as described [6-10]. They were suspended at about 20 mg of membrane protein/ml in membrane buffer (20 mM Hepes/40 mM citric acid/100 mM Tris gluconate/0.02% LiN3, supplemented to a total osmolarity of 560 mOsM with sorbitol and adjusted to pH 7.5 with Tris base) and stored in liquid nitrogen until the day of transport assay, as described [6-9]. Chloride transport was assayed by using ^36^Cl and a rapid filtration technique as described [8,9,11]. To test the effect of a peptide on proton-coupled Cl^- ^transport, BBM vesicles were mixed in the appropriate volume of membrane buffer in either the absence or presence of a given peptide. After preincubation for 5 min at 22°C, 5 μl aliquots were used to perform uptake measurements by mixing with 45 μl of transport buffer formed by the membrane buffer supplemented with 15 mM cis ^36^Cl, the amount of sorbitol necessary to obtain a total osmolarity of 660 mOsM, and adjusted to pH 5.0 with Tris base (final concentrations and pH in the incubation mixtures). Uncorrected initial (4 sec) uptake rates as a function of the inhibitor concentration (v = f [I] at constant substrate concentration) were fitted by non-linear least-squares regression analysis to Hill's equation. To perform each fit, the procedure of Fletcher and Powell as modified by van Melle and Robinson [12] was used. To test the fit of data to Hill's equation, we used the commercial program Stata (Integral Software, Paris, France). For statistical evaluation, fits were compared either within each given condition (F test) or between pairs of conditions (F' test), as described [6].

**Figure 1:**
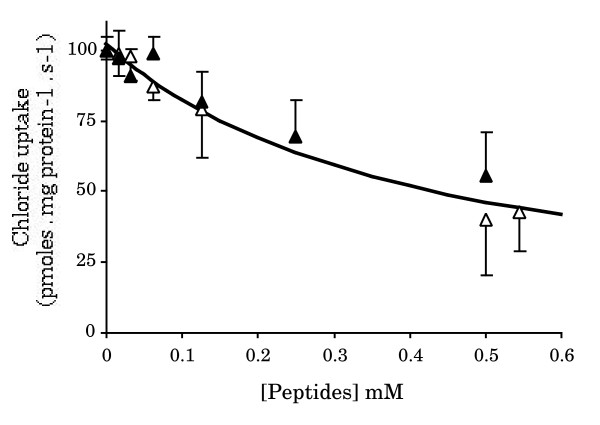
Dose-dependent effects of the NSP4_114-135 _and NV_464-483 _peptides on the initial rate of pH gradient-activated chloride uptake by rabbit intestinal BBM vesicles. Fixed amounts of BBM vesicles were mixed with variable amounts of peptide in the appropriate volume of membrane (Tris gluconate) buffer to give the indicated (final) peptide concentrations. After preincubation for 5 min at 22°C, aliquots were used to measure the initial rate of 15 mM chloride uptake in the presence of an initial pHo/pHi = 5.0/7.5 gradient. Symbols: (▲) for NSP4_114-135 _and (△) for NV_464-483_. Results are shown as absolute uptake rates in pmoles·mg membrane protein^-1^·s^-1 ^± standard deviation with 6 – 15 determinations per point. Because the two peptides gave statistically indistinguishable results, the two sets of data were pooled to obtain the overall fits in Table 1. The continuous line illustrates the best overall fit obtained by fixing the n_i _value to 1.

We earlier showed that an alkaline-inside pH gradient can furnish the energy necessary for the uphill transport of Cl^- ^across intestinal villi BBM purified from four week-old rabbits, indicating the presence of a Cl^-^/H^+ ^symporter in these vesicles [8,9]. To investigate the possible direct effect of each of these peptides on chloride influx, the initial Cl^- ^entry rates were quantified by using the v = f [I] approach in the presence of a pHo/pHi = 5.0/7.5 gradient (o (out) and i (in) indicate the extra- and intravesicular spaces, respectively). As illustrated in figure 1, both the NSP4_114-135 _and NV_464-483 _peptides caused a practically identical inhibition of proton-coupled Cl^- ^uptake across the BBM, approaching 55 % and 40 % inhibition, respectively, at 0.5 mM peptide. Analysis of the chloride results according to Hill's equation revealed that the two peptides gave statistically indistinguishable data (see F' test in Table 1). Therefore, the data were pooled and fitted again to obtain the overall fit given in Table 1 (line 3). To find the best integer value of the Hill number, the data were further fitted by fixing the ni value to either 1 or 2. According to the F value, one peptide-binding site per carrier unit is involved in the interaction with BBM (Table 1).

**Table 1:** Kinetic parameters used to compute the theoretical curve in FIG. 1.

	Kinetic parameter			F tests		
		
	V	Ki	ni	F	[df]	P
NV_464-483_	102 ± 8	0.34 ± 0.07	1.1 ± 0.3	0.307	[4, 34]	NS
NSP4_114-135_	100 ± 3	0.56 ± 0.07	1.1 ± 0.2	0.962	[4, 61]	NS
Overall fit	99 ± 3	0.43 ± 0.04	1.3 ± 0.2	0.332	[5, 101]	NS
Overall fit	102 ± 3	0.41 ± 0.04	{1}	0.637	[6, 101]	NS
Overall fit	94 ± 2	0.47 ± 0.02	{2}	1.279	[6, 101]	NS

Curve comparison				F'	[df ]	P
NSP4_114-135 _versus NV_464-483_				3.84	[3, 103]	NS

Kinetic parameters (± SD) for the function v = f [I] (at constant substrate concentration = 15 mM) were estimated by applying the following equation: v = V/(1 + {([I]/K_i_) n_i_}) where V is an apparent maximum velocity; K_i _is the apparent affinity constant for the inhibitor, I (the peptide) and n_i _is the Hill number. (See equation 2 in [6]). Units: V = absolute uptake rates in pmoles·mg membrane protein^-1^·s^-1 ^; K_i _= mM. For the F test, the degrees of freedom [df] for pure error and lack of fit, in that order, are indicated in brackets. P = NS (not significant to p < 0.01) means that, for each given curve, the data points do not differ statistically from the theoretical fit of the equation under study. For the F' test, the results were further compared by pairs where NS indicates that the two curves do not differ significantly from one another at p < 0.01. Because both peptides gave statistically indistinguishable data, the relevant data were pooled and fitted again to yield the indicated overall fit values. The symbol {} means that the n_i _value was fixed to an integer value before running the iteration. Further details in the text.

Taken together, the whole set of available results for peptide concentrations from 0.015 to 0.55 mM strongly supports the conclusion that the rotavirus NSP4_114-135 _peptide caused *in vitro *nonspecific inhibition of the Cl^-^/H^+ ^symporter across villus cell BBM. Similar conclusions have been reached with the rabbit BBM Na^+^-L-leucine symporter, further confirming nonspecific, lipophilic interactions of the amphipathic peptides with the membrane [6]. Clearly, the results indicate the existence of NSP4-lipid interactions with biological membranes, as could be expected from the presence of regions of high curvatures in intestinal BBM [13]. However, the interactions appear to be unimportant for the direct biological effects of NSP4, at least as concerns the rabbit BBM Cl^-^/H^+ ^and Na^+^-L-leucine symporters at the villus cell level. Whereas *in vitro *NSP4_114-135 _inhibits the Cl^-^/H^+ ^symporter, the situation in vivo is different. Because rotavirus accelerated both Cl^- ^influx and Cl^- ^efflux rates across villi BBM whereas it failed to stimulate Cl^- ^transport in crypt BBM [8,9], we conclude that NSP4 has no direct effect on either intestinal absorption or secretion of chloride.

Interestingly, the lack of direct effect of NSP4 on the BBM Cl^-^/H^+ ^symporter strengthens the hypothesis that NSP4 would trigger signal transduction pathways to enhance net chloride secretion at the onset of diarrhea [2,3,14]. Whether NSP4 or its cleavage product, NSP4_112-175_, after it is released from virus-infected cells [15], binds to an apical membrane receptor in villus enterocytes or crypt cells, or both, to activate intracellular second messengers is not known. Furthermore, the possibility that the secreted NSP4 – as most luminal enterotoxins – may reach the crypt region would seem to be unlikely [2]. Using human intestinal epithelial HT-29 cells, it was shown that exogenous addition of NSP4 induced intracellular calcium mobilization through phospholipase C (PLC) signaling [16]. The same signaling pathway was also found in rotavirus-infected Caco-2 cells [17]. Such PLC activation can lead to transient chloride secretion [18,19]. Also, NSP4-mediated Ca^2+ ^mobilization can lead to an activation of the nervous system (ENS) in the intestinal wall, and hence stimulated intestinal chloride secretion [2]. However, many details regarding the ENS-linked hypothesis of rotavirus-induced secretory diarrhea remain to be elucidated [14]. Obviously, more work will need to be performed before a clear understanding of the regulatory mechanisms of intestinal net chloride secretion during rotavirus and NSP4-mediated diarrhea can be achieved.
